# Early diagnosis of lung cancer: which is the optimal choice?

**DOI:** 10.18632/aging.202504

**Published:** 2021-02-11

**Authors:** Jing Ning, Tao Ge, Minlin Jiang, Keyi Jia, Lei Wang, Wei Li, Bin Chen, Yu Liu, Hao Wang, Sha Zhao, Yayi He

**Affiliations:** 1 Department of Medical Oncology, Shanghai Pulmonary Hospital, Tongji University Medical School Cancer Institute, Tongji University School of Medicine, Shanghai 200433, People’s Republic of China; 2 Tongji University, Shanghai 200433, People’s Republic of China

**Keywords:** LDCT, bronchoscopy, diagnosis, liquid biopsy, lung cancer

## Abstract

The prognosis of lung cancer patients with different clinical stages is significantly different. The 5-year survival of stage IA groups can exceed 90%, while patients with stage IV can be less than 10%. Therefore, early diagnosis is extremely important for lung cancer patients. This research focused on various diagnosis methods of early lung cancer, including imaging screening, bronchoscopy, and emerging potential liquid biopsies, as well as volatile organic compounds, autoantibodies, aiming to improve the early diagnosis rate and explore feasible and effective early diagnosis strategies.

## INTRODUCTION

According to Global Cancer Statistics, lung cancer has high incidence and mortality [1]. Most lung cancer patients are diagnosed at an advanced stage [2]. The reason is that most lung cancer patients have no obvious specific symptoms at the beginning of the disease, and many patients are at an advanced stage when they are definitely diagnosed [3]. Statistics show that the prognosis of lung cancer is closely associated with the clinical stage, which indicates that the early diagnosis can directly improve the prognosis of the patient [4]. Therefore, the selection of effective diagnosis and screening methods is quite crucial to improve the early diagnosis rate and prognosis of lung cancer.

## Imaging examination and screening for lung cancer

### Chest x-ray

It is not recommended to use chest x-ray to detect lung cancer at present, owing to *the difficulty of displaying* intrapulmonary lesions and small lesions. Furthermore, whether combined with sputum cytology examination or not, chest x-ray screening cannot reduce lung cancer disease-specific mortality [5, 6].

### Low-dose CT (LDCT)

LDCT is recommended as an early screening method for high-risk groups [7]. The National Lung Screening Trial (NLST) [8] recommended LDCT scan during lung cancer screening. The results illustrated that compared with chest x-ray, LDCT screening could reduce mortality by 20.0%. Furthermore, Agency for Healthcare Research and Quality (AHRQ) summarized 8,149 papers published from 2000 to the last quarter of 2012, which supported lung cancer early screening. Based on these findings, USPSTF, the American Cancer Society (ACS) and many other medical organizations recommended that LDCT screening should be considered first for patients who meet the NLST inclusion criteria [9–11]. In addition, the NCCN guidelines also suggested that LDCT was used to screen high-risk groups, but it was not recommended for low and moderate-risk groups [11, 12].

The advantages of LDCT include low radiation dose, fast scanning speed, and sensitivity comparable to CT. However, it not only shows a false positive rate (FPR) of 23.3% in NLST, which is similar to the results of other studies [8, 13, 14], but also has several disadvantages such as over-diagnosis, radiation exposure and high cost [7, 15, 16]. Therefore, the application of LDCT for early screening of lung cancer still has many problems to be overcome. For example, further clarify the screening time interval to balance the benefits and potential harm; accurately classify the high-risk population of lung cancer to avoid over-diagnosis and treatment; and what kind of model should be established to evaluate which types of lung imaging features are prone to develop into lung cancer. These are still urgent problems to be solved in the future.

### Positron emission tomography/CT (PET/CT)

PET/CT is an integrated fusion of PET and CT equipment and imaging, which can display accurate anatomical images and tissue metabolic function images. It has been widely used to identify the property and stage of tumors. Wang et al. [17] integrated 1330 patients with lung-occupying lesions in four clinical studies and found that PET/CT has higher sensitivity (98.7%) and higher specificity (58.2%) in distinguishing benign or malignant lung lesions. Yet, PET/CT also has a high FPR in lung cancer screening, and reducing FPR is still a dilemma in the early diagnosis of lung cancer through PET/CT [18]. In contrast with CT, PET/CT can enhance the accuracy of diagnosis of solitary pulmonary nodule (SPN) [19, 20]. The standard uptake value (SUV) of PET/CT reflects the metabolism and malignancy of diseased tissues under certain circumstances [21]. However, PET/CT also has its shortcomings. For instance, there are respiratory motion artifacts. FDG metabolism is not unique to tumors and the higher cost limits the application.

## Bronchoscopy and diagnosis for lung cancer

Nowadays, pathological diagnosis has been regarded as gold standard for diagnosing cancer. There are several methods for obtaining histological specimens, including bronchoscopy, ultrasound or CT-guided percutaneous lung biopsy. Among them, bronchoscopy has been developed rapidly and widely recognized in recent years. It not only expands the field of vision for diagnosis, but also improves the efficiency of diagnosis.

### White light bronchoscopy (WLB)

It is mainly used for early detection and diagnosis of central lung cancer, and the diagnosis rate can reach more than 95% in detecting high-grade dysplasia or worse. However, for some mucosal, submucosal early lesions and preneoplastic lesions, the diagnosis rate is very low (<30%) [22, 23].

### Autofluorescence bronchoscopy (AFB)

The operating principle of AFB is that different spectrum emerge in normal tissues, dysplasia and carcinoma *in situ* [24]. As an important means for early detection of bronchial premalignant lesions, some studies have proposed that compared with sputum cytology, the sensitivity of AFB to detect hyperplasia and metaplasia was higher. Regardless of the results of sputum cytology, AFB can be recommended to high-risk patients. Moreover, when combined with spiral computed tomography (SCT), sputum examination or WLB, AFB can obviously enhance the diagnosis rate of premalignant lesions and carcinoma *in situ* [22, 25, 26]. Nevertheless, due to the high cost of equipment and inspection, it has not yet been widely accepted.

### Narrow band imaging (NBI)

NBI is an imaging technique that can visualize vascular morphology and mucosal structure. A meta-analysis indicated that NBI has higher sensitivity (80%), specificity (84%) and diagnostic odds ratio (DOR 31.49%) than AFB in detecting premalignant airway lesions [27]. Another meta-analysis showed that NBI was also superior to WLB in detecting early and invasive lung cancer [28]. Besides, a prospective study suggested that dotted blood vessel visual pattern highly supported adenocarcinoma histology of lung cancer and tortuous vessels favored squamous cell cancer [29]. Therefore, when examining lung-occupying lesions with NBI, not only can the diagnosis rate be improved, but also the pathological type can be evaluated initially. So in the detection of early lung cancer, NBI can be used as an effective method.

### Endo-bronchial ultrasound (EBUS)

EBUS combined with a special aspiration biopsy needle can be used for real-time ultrasound guided transbronchial needle aspiration biopsy, namely EBUS-TNAB [30]. In a randomized controlled trial [31], when EBUS-TBNA was used as the initial examination method after CT scanning in patients with suspected lung cancer, it could provide accurate diagnosis and lymph node staging. Compared with traditional diagnosis strategies, EBUS-TNAB can shorten the time for treatment decisions and may improve the survival of lung cancer patients without increasing costs.

**Figure 1:**
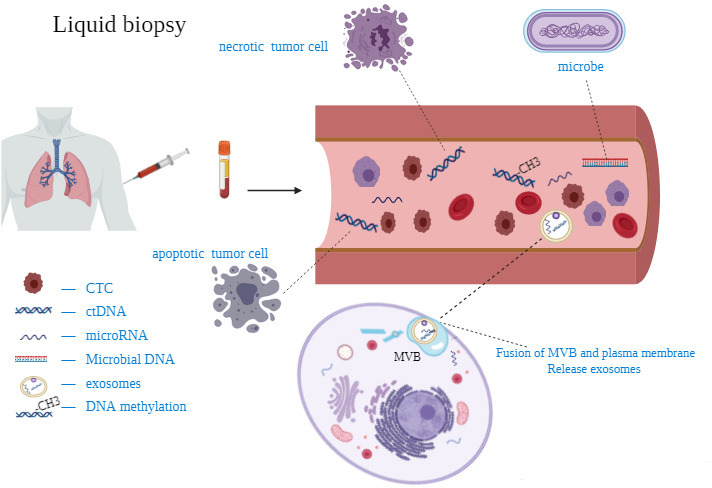
**Some studies on circulating miRNAs for diagnosis of lung cancer.**

Due to tumor heterogeneity, tissue biopsy just represents the state of the tumor at a certain moment and only a part of the malignant tumor, which cannot reflect the dynamic process of disease development [32]. Moreover, multiple biopsies are very invasive to patients, so new technologies need to be designed to improve this dilemma. Liquid biopsy can replace and complement this method [33, 34] (Figure 1).

## Liquid biopsy and screening for lung cancer

### Circulating tumor cells (CTCs)

CTCs is released into the peripheral blood from a primary cancer or metastasis [33]. The capture of CTCs is of great significance for the early detection, diagnosis, prognosis and monitoring of cancer, as well as understanding the basic biology of the metastatic process [35]. The content of CTC is extremely low (1-10 CTCs/1ml peripheral blood), which makes CTC separation and enrichment technology difficult. The CellSearch system was approved as the first and only method by FDA for CTC detection [36]. The CellSearch CTCs detection kit contains ferrofluid-based capture reagents and fluorescent immunoreagents. The ferrofluid-based reagent is a particle with a magnetic core, the surface of which is coated with antibodies that recognize EpCAM antigen. EpCAM is a CTC-specific antigen. Therefore, the magnetic particles can capture CTC. After immunocapture and enrichment, fluorescent reagents are used to identify CTCs and CTCs counts. EpCAM (+) CK (+) DAPI (+) CD45 (-) cells are defined as CTCs. In addition, there are other common ways to detect CTCs (Table 1).

**Table 1:** Comparison of common CTCs isolation and enrichment techniques.

**Methods**	**Representative examples**	**Advantages**	**Disadvantages**
Coated anti-EpCAM antibody	Cellsearch (FDA approved)	High repeatability and specificity	Due to EMT, epithelial specific antigens were lost, resulting in loss of capture efficiency
Size-Based selection	Isolation by size of epithelial tumor cells (ISET) [37]	Don’t rely on EpCAM antigen on the surface of CTCs	Don’t detect CTCs with a very small size
Density-Based selection	Oncoquick [38]	Preserve cell viability	Low sensitivity and unstable, Missing rare CTCs
Microfluidics	CTC-CHIP [35]	High sensitivity and high detection rate	Lack of clinical verification
immunomagnetic	Magnetic Activated Cell Sorting (MACS) [39]	Preserve cell viability and easy to count	Adsorption of non-specific cells containing target markers leads to decreased sensitivity

Lou et al. [40] and Zhang et al. [41] suggested that CTCs could be detected in the lung cancer, especially stage I to IIIA. He et al. [42] evaluated that the CTC detection rate (CTC>1) of stage I and stage II lung cancer was as high as 62.5% by using CellCollector and there were 71.6% similarities between CTCs and tumor tissues in gene mutation site of TP53, FGFR1, HER2, PDGFRA and CFS1R. It is the first time to reveal the molecular and genetic variation characteristics of early lung cancer CTC, and provides new data support for the clinical application of CTC as a molecular diagnosis of early lung cancer.

In summary, as a sign of early diagnosis, CTCs also have certain limitations. Reasonable and effective enrichment methods are the most important and urgent problems which need to be overcome. The main challenge is to obtain a sufficient number of CTCs in optimal conditions for further evaluation. The technology for assessing the molecular characteristics of CTCs is still evolving and how to standardize them for clinical application need to be studied.

### Circulating tumor DNA (ctDNA)

The DNA, released by necrotic or apoptotic cells into the blood, is called circulating free DNA (cfDNA). In the blood of tumor patients, part of the cfDNA from dead tumor cells is defined as ctDNA [43]. Normally, ctDNA accounts for only 0.01-1% of cfDNA and is released from multiple tumor regions, which can overcome tumor heterogeneity [44]. It is reported that plasma is a good source of ctDNA [45]. The ctDNA can be quantitatively detected by the technology based on the four main components of bead, emulsion, amplification and magnetic, while the CAPP-seq technology for personalized cancer analysis through deep sequencing has changed the diagnostic mode of ctDNA [46, 47].

So far, ctDNA is the best material to obtain the diagnosis, prognosis and predictive information of tumor-related changes in the blood of cancer patients [48].The study by Szpechcinski et al. [49] showed that the level of plasma ctDNA in patients with non-small cell lung cancer (NSCLC) were not only higher than that in healthy people, but also higher than those with chronic respiratory inflammatory diseases. Liang et al. [50] combined ctDNA and DNA methylation to distinguish lung cancer and benign nodules. The sensitivity and specificity of the method for distinguishing malignant tumors and benign lesions were 79.5% (63.5%~90.7%) and 85.2% (66.3%~95.8%). The method of identifying stage IB (sensitivity =85.7%) lung cancer was also better than stage IA (sensitivity =75.0%). Moreover, Chen et al. [51] measured ctDNA and tumor tissue DNA (tDNA) in patients with stage I to III NSCLC by targeted sequencing. They founded frequent driver mutations in ctDNA and tDNA, which confirmed the feasibility of peripheral blood ctDNA detection in early detection of NSCLC gene mutations. However, Sozzi et al. [52] reported that compared with cancer-free subjects, the DNA baseline levels of cohort that developed cancer during the 5-year period (n=38) was no significant difference (AUC =0.496), just slightly higher than the baseline at the time of diagnosis. But high levels of ctDNA were more likely to develop lung cancer in individuals who smoke heavily in longitudinal contrast.

Although ctDNA analysis provides a viable option for early diagnosis of lung cancer, the existing techniques still cannot conquer the difficulties of sensitivity analysis. In addition, there are many problems that need to be solved, including lack of reliable threshold value, the required purity of ctDNA, the low proportion of ctDNA reflecting all genetic mutations [33].

### Microbial DNA (mbDNA)

Multiple studies have shown that microorganisms were closely implicated to tumor progression and primary drug resistance in some cancers [53–55]. Recently, Poore et al. [56] studied the microbial sequencing of 18,116 samples from 10,481 patients with 33 cancer types reported in the TCGA database. These researchers also analyzed the whole genome sequence of 4831 samples, as well as RNA sequencing data from 13,285 tumor samples, normal tumor-adjacent tissue, blood samples and matched tissues from non-cancer individuals. The sequences of microbial sources were quickly screened by Kraken software, and used machine learning methods (ML) for microbial reads. For stage Ia–IIc cancers whose genome variants cannot be detected by ctDNA, mbDNA has stronger predictive ability. In order to further verify the effectiveness of these results, patients with prostate (n=59), lung (n=25) or melanoma (n=16) and healthy volunteers (n=69) were selected as samples. In addition to the smallest melanoma cohort, a high degree of distinction was found in paired and multiclass comparisons between normal samples and cancer types. This means that the new type of microbiome-based cancer diagnostic tool can complement existing ctDNA detection methods to detect and monitor cancer.

Unique microbial DNA signatures are found in the tissues and blood samples of most cancer patients, which can identify the presence and type of cancer. Compared with ctDNA, one of the advantages of cancer detection based on circulating microbial DNA is its diversity among different body parts. This model performs well in differentiating stage I and IV tumors of colon adenocarcinoma, gastric adenocarcinoma, and renal clear cell carcinoma. However, there are still many problems to be solved in the field of mbDNA. For example, in the process of preparing DNA, microbial contamination is a problem we must pay attention to, how to identify whether mbDNA is a normal aging change or a tumor-related change [57].

### MicroRNA(miRNA)

MiRNAs is a class of endogenous non-coding small single-stranded RNA (19~24 nucleotides), originally found in nematodes [58]. MiRNA is released into the blood through some mechanisms, including passive leakage from broken cells in damaged tissues, apoptotic cells and process of inflammation and metastasis; active secretion by microvesicles and microvesicle-free miRNAs through different stimuli [59]. It degrades the mRNA by binding to the 3'UTR of the target mRNA or mediating post-transcriptional silencing through protein translation inhibition [60]. Compared with mRNA, its high stability and repeatability are its unique features. The corresponding resistance is also shown after many freeze-thaw cycles [59, 61]. Thence, researchers have been devoted to building reliable diagnostic tools with miRNA.

Lawrie et al. [62] demonstrated that high expression of MIRN21 is correlated to relapse-free survival (P=0.05). Hereafter, Zen et al. [63] proposed that circulating miRNAs better reflected all aspects of disease than tissue miRNAs. To date, using a panel of miRNAs to detect early-stage lung cancer is more common than using single miRNA (Table 2).

**Table 2:** Some studies on circulating miRNAs for diagnosis of lung cancer.

**miRNA**	**TNM stage**	**Source**	**Detection method**	**Performance**	**Details**
miR-429,miR-205, miR-200b,miR-203,miR-125b,miR-34b	I-IV	serum	qRT-PCR	Sen=88% Spe=71% AUC=0.88 (I/II) AUC=0.89 (I/V) Threshold value=0.37	NSCLC (n=100) [64] non-cancer (n=58)
miR-146a,miR-222,miR-223	I-III	serum	qRT-PCR	AC vs HD: Total AUC=0.951 Sen=84.35% Spe=90.83% Acu=87.27% Cutoff value = 0.5015	Training set [65] AC (n=40) HD (n=40)
				In AC AUC=0.942 (I) AUC=0.968 (II) AUC=0.954(III)	Validation set AC (n=120) HD (n=120)
miR-21,miR-145, miR-155	I-III	plasma	qRT-PCR	Training set LC vs HS Total AUC=0.847 Sen=69.4% Spe=78.3% Cutoff value: 1.310, 0.4950,0.01535	Training set [66] LC (n=62) HS(n=60)
				Validation set LC vs HS Total AUC=0.841 Sen=76.5% Spe=80.0%	Validation set LC (n=34) BPN (n=30) HS (n=32)
miR-146b,miR-205,miR-29c, miR-30b	I-III	serum	qRT-PCR	Training set AUC=0.99 Sen=93.65% Spe=93.33% Acu=95.00% Cutoff value = 0.5606	Training set [67] NSCLS (n=63) HS (n=15)
				Validation set AUC=0.93 Sen=78.13% Spe=95.38% Acu=89.69%	Validation set NSCLS (n=65) Cancer-free (n=32)
				In NSCLC AUC=0.96(I) AUC=0.95(II-III)	
miR-1254 MALAT1 miR-485-5p miR-574-5p	I-IV	serum	qRT-PCR	AUC=0.864/0.848/ 0.878/0.732(I/II/III /IV) Sen=94.6%/89.7%/ 94.9%/66.7%(I/II/III /IV) Spe=75.7%(I/II/III/ IV)	Training set [68] NSCLS (n=36) HS (n=36)
					Validation set NSCLS (n=120) HS (n=71)

Abbreviation: Sen: sensitivity; Spe: specificity; Acu: accuracy; AC: lung adenocarcinoma; HD: healthy donor; HS: healthy smoker; LC: lung cancer; BPNs: benign pulmonary nodules; SCC: squamous cell carcinoma.

The World Conference on Lung Cancer 2019 reported the results of a clinical trial called BioMILD [69], which recruited 4119 participants. This trial combined LDCT scanning with miRNA testing to screen the high risk groups of lung cancer and implied that combining miRNA with LDCT examination could greatly enhance early diagnosis efficiency.

Non-invasiveness and stable nature of circulating miRNA make it a potential tool as a biomarker for diagnosing cancer. Nevertheless, the limitation of miRNA is reflected in the inconsistency that the selection of internal reference genes for quantitative detection. Moreover, miRNAs from different sources, such as tissues, plasma and serum, lack a standardization protocol during the separation and extraction process. Therefore, standardization is a problem that should be solved.

### Circulating exosomes

Exosomes are intracellular vesicles with a diameter between 30 nm and 100 nm. Fusion of multivesicular bodies with plasma membrane induces the release of exosomes from multivesicular bodies into the extracellular space [70]. It can be detected from various body fluids, comprising plasma, saliva, urine, breast milk, pleural effusion, cerebrospinal fluid, semen, and carries biological information such as protein, microRNA, mRNA, DNA [33, 70, 71]. It may promote invasion, immune escape and chemotherapy resistance of lung cancer [72]. Researchers are gradually paying attention to the potentiality of exosomes as a diagnostic biomarker.

Among them, there are many studies on miRNAs or proteins derived from circulating exosomes. Exosome miRNA (let-7b/let-7e/miR-23a-3p/miR-486) profiles in plasma determined by NGS technology can identify patients with stage I NSCLC (sensitivity= 80.3%, specificity= 92.3%, AUC=0.899) [73]. Li et al. [74] proposed that Exo-Gas5 (growth arrest-specific transcript 5) also has a similar effect as above. Moreover, when jointly evaluated with CEA, AUC value up to 0.929. In addition, when Exo-Gas5 was used alone to identify patients with stage I NSCLC, AUC value even reaches 0.822. Apart from these, more studies have shown that exosomes might be a candidate to develop highly sensitive, non-invasive and effective biomarkers for early NSCLC diagnosis [75].

Due to its nano-size, efficient extraction and purification protocol are still a big problem [33]. Yet it also has the characteristics of vesicle coating and stable properties, which can be acted as a natural drug carrier to broaden the prospect of exosomes in the treatment of lung cancer after definite diagnosis [76]. In short, exosomes are an indispensable development trend not only in early diagnosis but also in clinical pharmaceutical transformation.

### DNA methylation

DNA methylation refers to the methyl group covalently integrate to cytosine of the cytosine phosphate-guanine (CpG) dinucleotide in the DNA genome under the action of DNA methyltransferase (DNMT) [77]. Some areas with a length of 0.5 to 4kb have high CpG density, which are called CpG islands (CGIs), and most of them are located at the 5'end of the gene. Under normal circumstances, CpG dinucleotides in CGIs remain unmethylated [78]. In patients with malignancy, the CGIs of anti-oncogene promoter was abnormally hypermethylated, and the normal genome level showed hypomethylation [79]. Abnormal DNA methylation cause the increase of chromosome rotation, the silencing and loss of expression of anti-oncogene, which ultimately leads to tumorigenesis [80, 81]. Previous studies have proved that early recurrence of curative stage I lung cancer and early lung cancer have specific DNA methylation [82]. Thus, abnormal methylation of DNA can become a biomarker for diagnosis and monitoring of lung tumors.

Ma et al. [83] utilized quantum dots-based fluorescence resonance energy transfer (QDs-FRET) technique to quantitatively analyze the methylation levels of PCDHGB6, HOXA9 and RASSF1A in lung cancer tissues and specimens. It was found that compared with tumor tissues, bronchial brush specimens have a slightly weaker ability to detect early cancer. In 50 NSCLC subjects with stage I to II, the total sensitivity (92%) and specificity (100%) are higher than single gene (68%, 80%, 64%), but the sensitivity to identify stage II NSCLC is better than stage I NSCLC (100% vs 83%). Therefore, this method for detecting DNA methylation can act as a potential non-invasive clinical diagnosis tool for early stage cancer. Besides, peripheral blood and sputum are an ideal sample for detecting abnormal methylation in early lung cancer [84, 85]. Liu et al. [86] took subjects with suspicious nodules on CT images as the research object, and found that methylation detection of 6 genes (CDO1, TAC1, HOXA7, HOXA9, SOX17, ZFP42) in plasma and urine was significantly related to the diagnosis of NSCLC. Based on this research, it further confirmed the effectiveness of DNA methylation as an auxiliary test for lung cancer CT screening.

In conclusion, the research on DNA methylation-related issues in NSCLC helps to understand the pathogenesis at the molecular level, and also provides potential hope for the early diagnosis, outcome and treatment of NSCLC. However, there are still some problems in the application of DNA methylation as molecular marker in clinical diagnosis of lung cancer. First of all, there is a lack of uniform testing standards [79]. Secondly, as a tumor marker, the single gene positive rate is low. Last, the specificity is not high. All of these problems need to be ameliorated.

## Volatile organic compounds (VOCS) and screening for lung cancer

In recent years, the detection of VOCs has the advantages of rapid, non-invasive, convenient, high sensitivity and good repeatability [87]. Exhaled breath contains a lot of substances, most of which are VOCs [88]. As a new diagnostic method, it has become a research hotspot. For example, Zhong et al. [89] constructed a disposable colorimetric array from diverse chemo-responsive colorants. Through chemical interaction with VOCs, these arrays could distinguish 20 different VOCs associated with lung cancer during exhalation with an accuracy of at least 90%. Li et al. [90] developed doping-assisted low-pressure photoionization mass spectrometry (DA-LPPI), proving the effectiveness of dichloromethane DA-LPPI technology in the detection of lung cancer-related polar VOCs.

Now, there are many types of VOCs and their sources are complex. The main difficulty is that many test techniques and methods used in sample collection, processing, storage and testing still lack uniform standards [91]. Therefore, although there are still great challenges in converting to clinical applications, the huge prospects for lung cancer detection are undeniable.

## Autoantibodies and screening for lung cancer

Another clinical trial, ECLS, was also reported in World Conference on Lung Cancer 2019 and enrolled 12208 high-risk patients. EarlyCDT test is a novel blood test based on lung cancer autoantibodies. Patients receive EarlyCDT blood test. If there is a positive result, they will receive x-ray and CT scan, which is designated as the experimental group. Among the people who received EarlyCDT examination and continued to develop lung cancer in the next two years, 41.1% of patients were diagnosed in the early stages (stage I and II) of the disease, compared to 26.8% in the control group. After 2 years of follow-up in subjects randomized to participate in the EarlyCDT trial, it was found that the incidence of advanced lung cancer was reduced by 36%. The study also showed that the patients undergoing EarlyCDT testing had a subtle trend of lower lung cancer-related mortality within two years than the control group. This study proves that EarlyCDT has a strong application prospect in the clinical early diagnosis of lung cancer [92].

## Summary

Among the above-mentioned early screening and diagnostic methods of lung cancer, x-ray screening is not recommended. LDCT has obvious advantages and is the most promising imaging method in early screening of lung cancer. Bronchoscopy has a greater advantage in direct vision of intraluminal lesions and can be used as a diagnostic tool. Liquid biopsy, VOCs and special tumor autoantibodies detection are simple and non-invasive. Especially liquid biopsy, it is a hot spot and the direction of future research in early diagnosis of lung cancer in recent years. However, there is currently no clear threshold and standard operating protocol for liquid biopsy, and tumor stage cannot be evaluated like CT. Therefore, the combination of LDCT and liquid biopsy to diagnose lung cancer or develop a reasonable diagnosis method may achieve early detection, early diagnosis and early treatment, thereby benefiting lung cancer patients. Besides, more large-scale research needs to be carried out.
